# Supplementary material for: Polyhydroxyalkanoate Decelerates the Release of Paclitaxel from Poly(lactic-co-glycolic acid) Nanoparticles
Source: Pharmaceutics. 2022 Aug 2;14(8):1618. doi: 10.3390/pharmaceutics14081618 (PMC9416746; doi:10.3390/pharmaceutics14081618)
Supplement: Supplementary file 1 [file pharmaceutics-14-01618-s001.zip › pharmaceutics-1787070-supplementary.pdf]

Supplement

Polyhydroxyalkanoate decelerates the release of paclitaxel from poly(lactic-co-glycolic acid) nanoparticles

Si Yeong Lee [1], So Yun Kim [1], Sook Hee Ku [2], Eun Ji Park [3], Dong-Jin Jang [4], Sung Tae Kim [1,5,*] and Seong-Bo Kim [6,*]

1. Transmission electron microscope (TEM)

To obtain negatively stained TEM images, 5 μl of each sample (as purified) was applied to carbon-coated grids that had been glow-discharged (Harrick Plasma, U.S) for 1 min in air. The grids were then negatively stained using 1% uranyl acetate [1]. The prepared grids were observed on a Tecnai 10 transmission electron microscope (FEI, Instrumentation was used in the Kangwon Center for Systems Imaging) equipped with a lanthanum hexaboride (LaB_6, FEI) cathode operating at 100 kV. Images were recorded on a 2K x 2K UltraScan CCD camera (Gatan). The instruments were installed at Kangwon Center for Systems Imaging, Chuncheon, Republic of Korea.

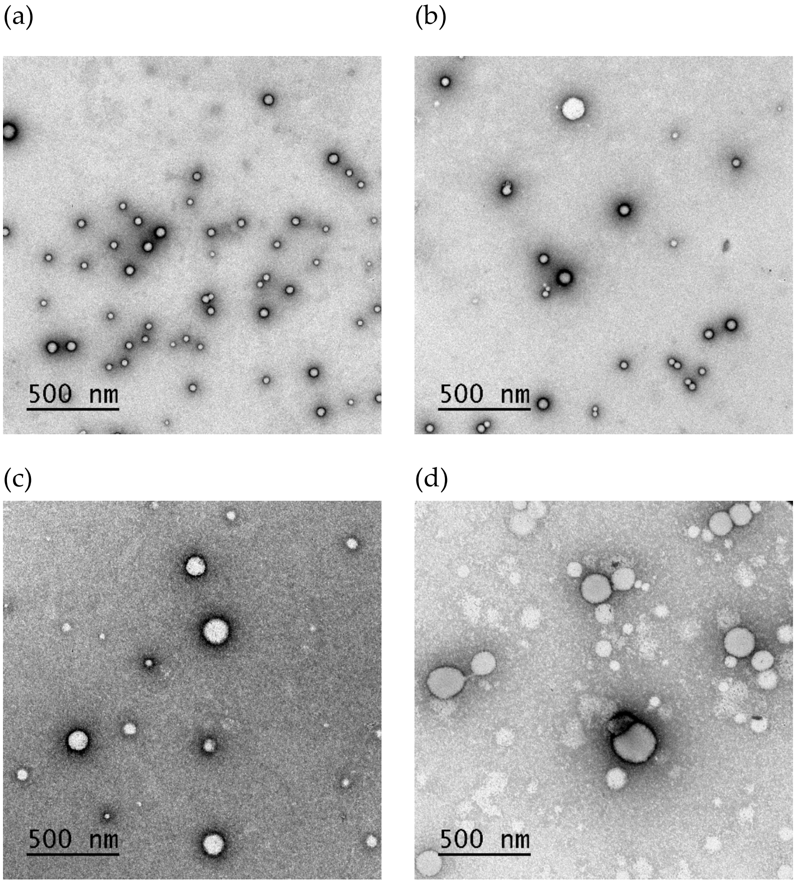

Figure S1. TEM images of NPs (**a**) NP1, (**b**) NP2, (**c**) NP3, (**d**) NP4.

2. Physicochemical characterization of raw materials

2.1. Fourier-transform infrared (FT-IR) spectroscopic analysis

The spectra of PLGA corresponded to C-H stretching vibration at 2,995.48 cm^{-1} and the peak at 1,754.72 cm^{-1} corresponded to carbonyl (C=O) ester bond stretching vibration. [2,3] And the spectra of PHA also was similar with that of NP4 (PHA nanoparticle). The representative band at 1,725.04 cm^{-1} corresponded to carbonyl (C=O) ester bond stretching vibration and the other peak was revealed at 2,969.26 cm^{-1}. [4,5]

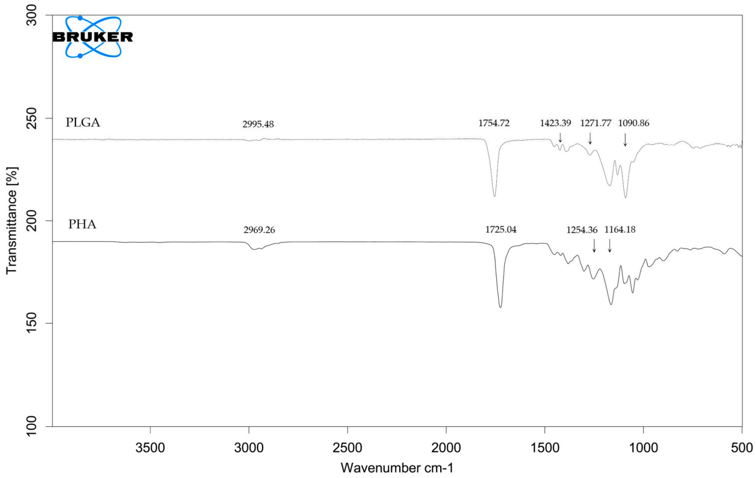

Figure S2a. FT-IR spectra of PLGA and PHA. The major peaks are indicated for each spectrum of PLGA and PHA, respectively.

2.2 Differential scanning calorimetric (DSC) analysis

The endothermic peak in PLGA was observed at 47.19°C, corresponding to the glass transition temperature (Tg) [6]. The PHA showed no peaks related to crystallinity and melting temperature, indicating amorphous state in a given range of condition [7]. Also, there was no significantly different peaks in XRD analysis compared to NPs composed of PLGA and PHA.

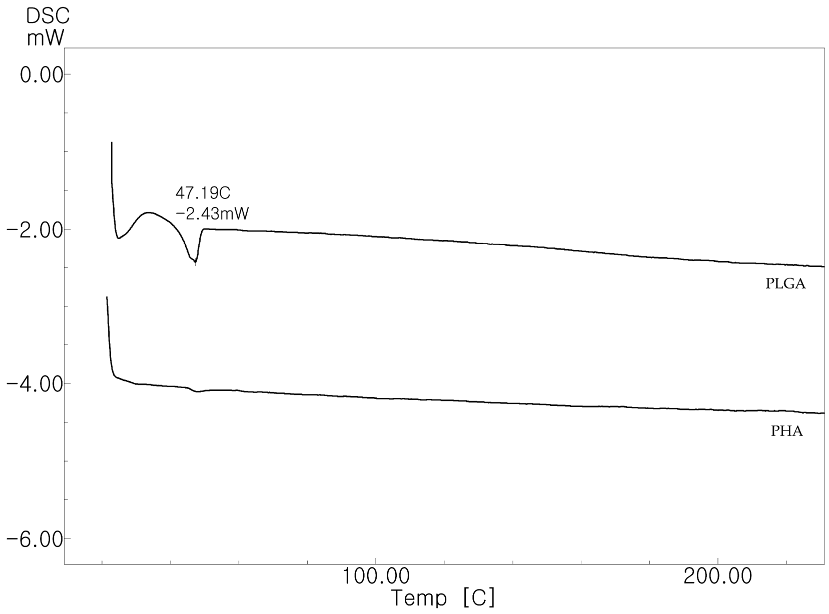

Figure S2b. DSC analysis of PLGA and PHA. The major peaks are indicated for each DSC curve of PLGA and PHA, respectively.

2.3. X-ray diffraction (XRD) analysis of NPs

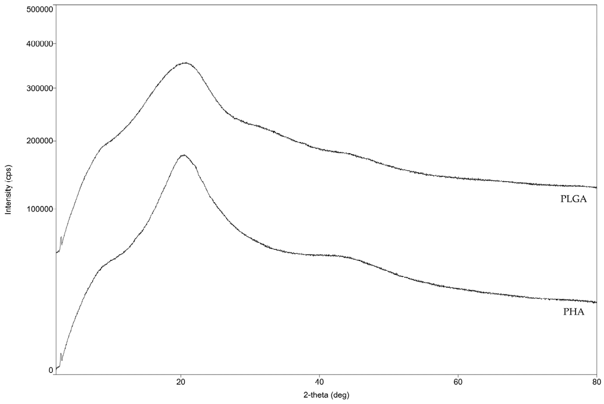

Figure S2c. XRD spectrum of PLGA and PHA, respectively.

3. Storage stability of NPs

The NPs were stored at 4°C, and their size and zeta potential values were measured to evaluate particle stability in various conditions such as saline and serum-containing saline, as shown in FigureS3. None of the NPs showed any significant change with respect to the size during the given period.

(a)

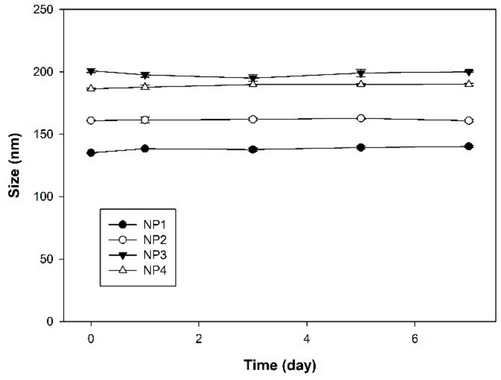

(b)

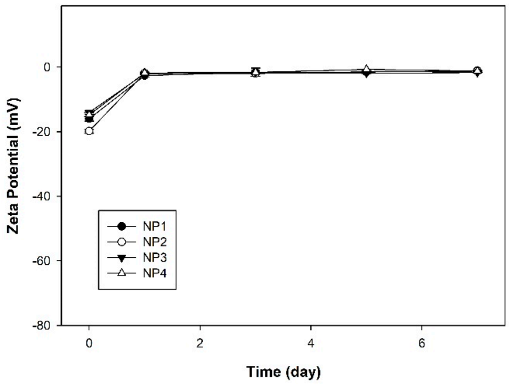

(c)

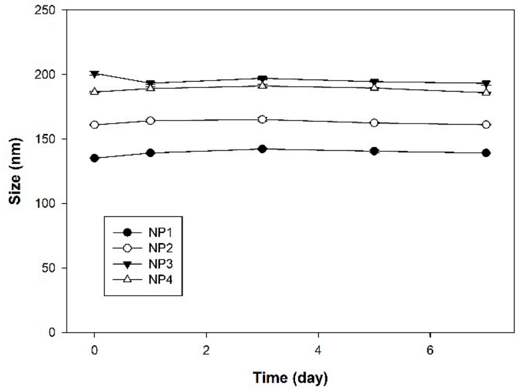

(d)

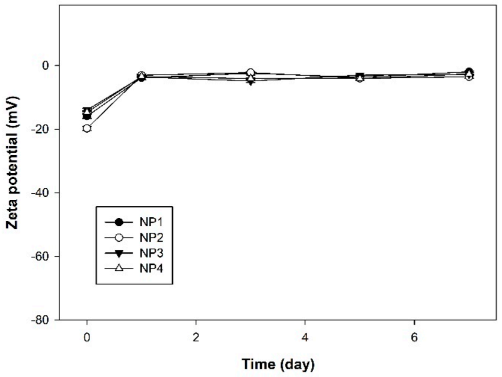

Figure S3. Storage stability of NPs in saline (a, b) and 10% fetal bovine serum-containing saline (c, d) at

4°C for 1 week. Both size and zeta potential (ζ) values of NPs were monitored: (a) changes of size in saline, (b) changes of zeta potential values in saline, (c) changes of size of NPs in 10% fetal bovine serum-containing saline, (d) changes of zeta potential values in 10% fetal bovine serum-containing saline.

4. PTX release kinetics

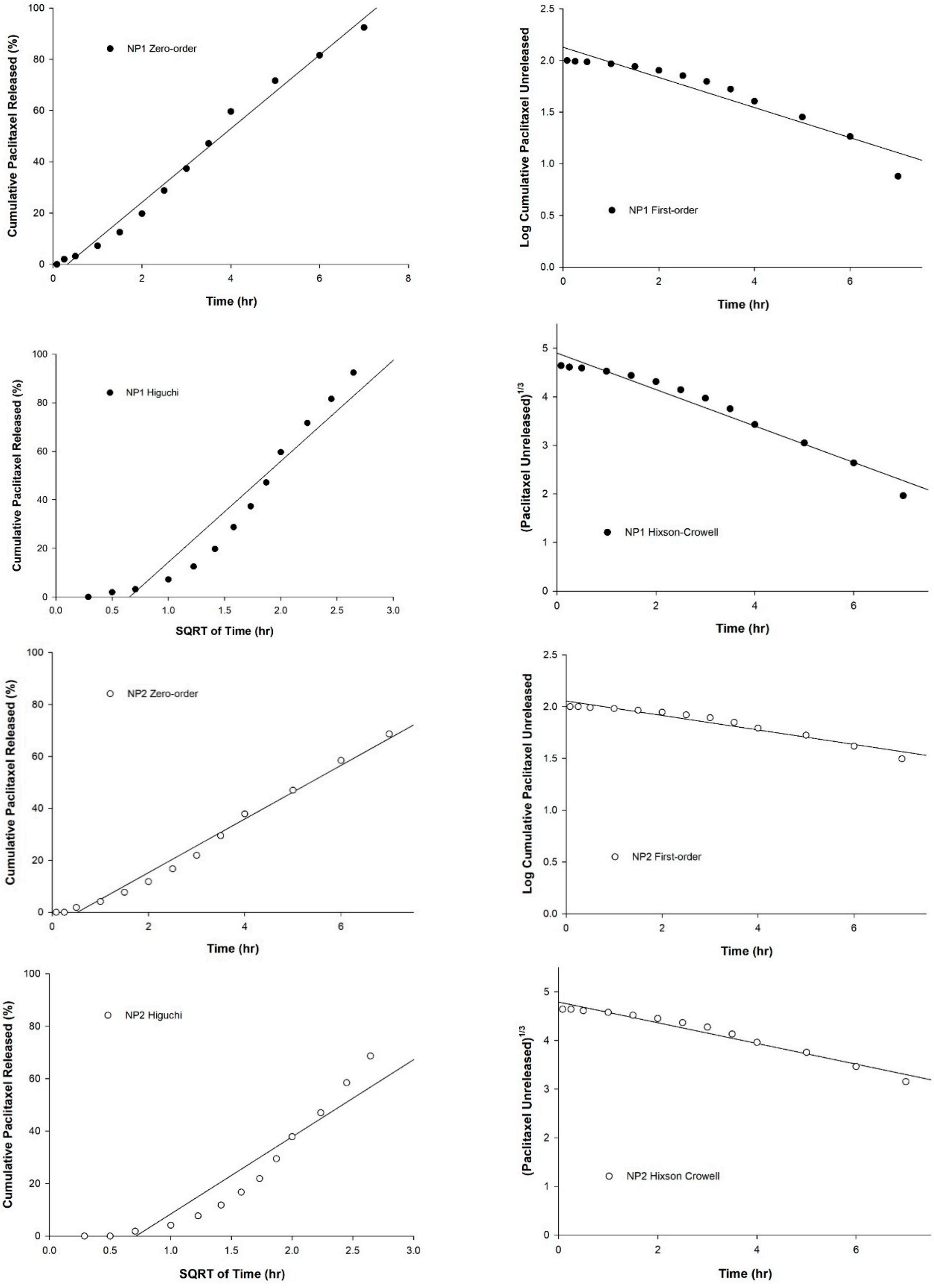

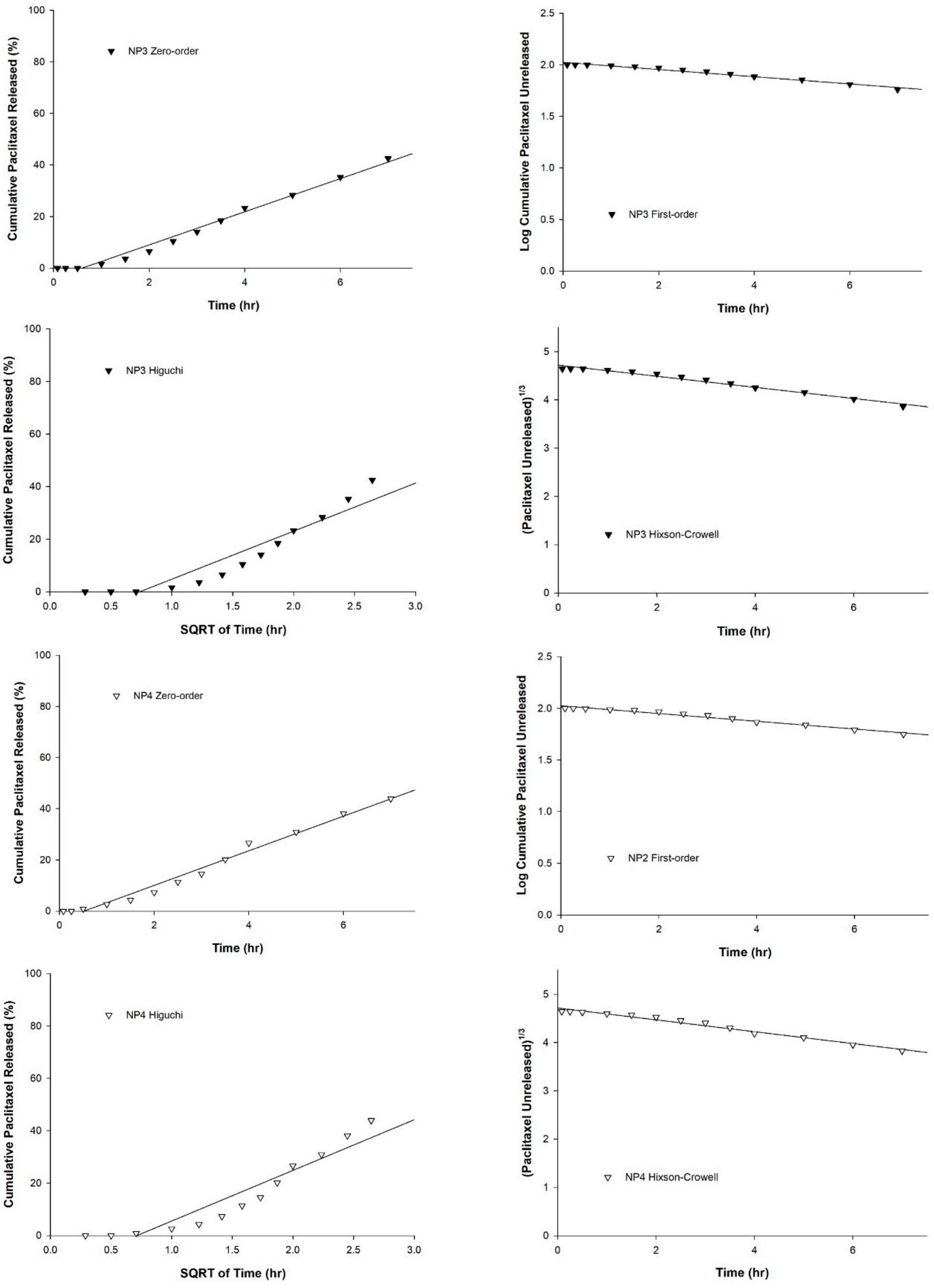

Figure S4. PTX release data fitted to various kinetic models (zero-order, first-order, Higuchi, and Hixson-Crowell) of NPs obtained from dissolution studies in buffer (pH 7.4).

References

[1] Kim, H.; Bharda, A. V.; Moon, J. C.; Jeoung, D.; Chung, J. M.; Jung, H. S. Microscopic studies on severing properties of actin-binding protein: its potential use in therapeutic treatment of actin-rich inclusions. *J. Anal. Sci. Technol.* **2021,** *12(1),* 1-6; https://doi.org/10.1186/s40543-021-00305-2

[2] Altındal, D. Ç.; Gümüşderelioğlu, M. Melatonin releasing PLGA micro/nanoparticles and their effect on osteosarcoma cells. *J. Microencapsul.* **2016,** *33(1),* 53-63; https://doi.org/10.3109/02652048.2015.1115901

[3] Silva, A. T. C. R.; Cardoso, B. C. O.; e Silva, M. E. S. R.; Freitas, R. F. S.; Sousa, R. G. Synthesis, characterization, and study of PLGA copolymer in vitro degradation. *J. Biomater. Nanobiotechnol.* **2015,** *6(01),* 8; https://doi.org/10.4236/jbnb.2015.61002

[4] Samrot, A. V.; Samanvitha, S. K.; Shobana, N.; Renitta, E. R.; Senthilkumar, P.; Kumar, S. S.; Abirami, S.; Dhiva, S.; Bavanilatha, M.; Prakash, P. The synthesis, characterization and applications of polyhydroxyalkanoates (PHAs) and PHA-based nanoparticles. *Polymers* **2021,** *13(19),* 3302; https://doi.org/10.3390/polym13193302

[5] Volova, T.; Zhila, N.; Shishatskaya, E.; Mironov, P.; Vasil'Ev, A.; Sukovatyi, A.; Sinskey, A. The physicochemical properties of polyhydroxyalkanoates with different chemical structures. *Polym. Sci. – A* **2013,** *55(7),* 427-437; https://doi.org/10.1134/s0965545x13070080

[6] Ayyoob, M.; Kim, Y. J. Effect of chemical composition variant and oxygen plasma treatments on the wettability of PLGA thin films, synthesized by direct copolycondensation. *Polymers* **2018,** *10(10),* 1132; https://doi.org/10.3390/polym10101132

[7] Sharma, P. K.; Munir, R. I.; Blunt, W.; Dartiailh, C.; Cheng, J.; Charles, T. C.; Levin, D. B. Synthesis and physical properties of polyhydroxyalkanoate polymers with different monomer compositions by recombinant Pseudomonas putida LS46 expressing a novel PHA synthase (PhaC116) enzyme. *Appl. Sci.* **2017,** *7(3),* 242; https://doi.org/10.3390/app7030242
